# A Colorimetric Immunoassay Based on g-C_3_N_4_@Fe_3_O_4_ Nanocomposite for Detection of Carcinoembryonic Antigen

**DOI:** 10.1155/2022/6966470

**Published:** 2022-01-28

**Authors:** Yanling Zhao, Yanfei Wen, Xing Hu, Bing Zhang

**Affiliations:** ^1^Shanxi Medical University, Taiyuan 030001, China; ^2^College of Biomedical Engineering, Taiyuan University of Technology, Taiyuan 030024, China

## Abstract

We proposed a colorimetric immunosensor based on g-C_3_N_4_@Fe_3_O_4_ nanocomposite-mediated transformation strategy for sensitive detection of carcinoembryonic antigen (CEA). The g-C_3_N_4_@Fe_3_O_4_ nanocomposite was synthesized and characterized by the scanning electron microscope (SEM), energy dispersive X-ray spectra (EDX), X-ray powder diffraction (XRD), and Fourier transform infrared spectroscopy (FTIR). Fe^3+^ derived from g-C_3_N_4_@Fe_3_O_4_ nanocomposite could combine with sodium salicylate to form purple complex products. Based on this color development, the sandwich colorimetric immunoassay was built by utilizing g-C_3_N_4_@Fe_3_O_4_ nanocomposite as nanolabels on the microplate. With the increasement of CEA concentration, the purple color showed a gradient change. Under optimal conditions, the linearity range is 0.001–50 ng/mL with the detection limit of 0.35 pg/mL for CEA. More importantly, the colorimetric immunoassay has good selectivity, specificity, repeatability, and stability.

## 1. Introduction

The level of carcinoembryonic antigen (CEA) is related to many diseases such as breast cancer, lung cancer, colon cancer, rectal cancer, and so on [1]. CEA as broad-spectrum tumor marker is important for early diagnosis and treatment of cancer [2, 3], and the methods for CEA detection are of great concern [4–6]. In recent years, great efforts have been made to develop various immunoassay methods, such as photoelectrochemical [7], fluorescence [8], chemiluminescence [9], surface-enhanced Raman scattering [10], enzyme-linked immunosorbent assay (ELISA) [11], colorimetry [12], and so on. In these methods, colorimetric immunoassay has attracted much attention due to its simplicity and conveniency [13, 14].

A key challenge for the development of colorimetric immunoassay is to transform the detection event into color change. Various strategies have been developed for this purpose, such as aggregation-based colorimetric immunoassay, lateral-flow colorimetric immunoassay, enzyme-mediated colorimetric immunoassay, and light-enabled colorimetric immunoassay [15–18]. Aggregation-based colorimetric immunoassay and colorimetric lateral-flow immunoassay are user-friendly, fast, and cost-effective, which are usually employed for fast on-site analysis. Enzyme-mediated colorimetric immunoassay and light-enabled colorimetric immunoassay need the participation of bioenzyme and laser, which is expensive and complex to operate. Hence, it is meaningful to develop the novel colorimetric method to enhance practicability. Various chemical color reactions have been developed and utilized in the field of pharmaceutical analysis and environmental analysis [19–21]. The chromogenic reaction between the phenolic hydroxyl group and Fe^3+^ is often used to identify the presence of phenols, which forms a purple complex [22].

In order to improve the sensitivity of chromogenic reaction and its application in immunoassay, nanomaterials with a specific interfacial effect and small-size effect have been employed [23–25]. Carbon nitrogen (C_3_N_4_), as a kind of metal-free material, has attracted much attention in biosensing filed due to its easy preparation, good biocompatibility, and high specific surface area. A series of C_3_N_4_-based composite nanomaterials including WO_3_/g-C_3_N_4_/MnO_2_, Ni-doped SnO_2_/g-C_3_N_4_, g-C_3_N_4_-COOH/ZnSe, and so on are synthesized and used in analytical applications [26–29]. Zhang et al. synthesized graphitic carbon nitride nanosheets-supported palladium nanosheets composite (Pd/g-C_3_N_4_) with oxidase-like activity for acetylcholinesterase (AChE) activity detection [30]. Ding et al. realized tumor marker detection using ternary GO-C_3_N_4_-AgBr heterojunction nanophotocatalyst [31].

In this study, g-C_3_N_4_@Fe_3_O_4_ nanocomposites were prepared as nanolabels to build colorimetric immunoassay for CEA detection. Under acidic conditions, g-C_3_N_4_@Fe_3_O_4_ generates a mass of Fe^3+^, which reacted with sodium salicylate and formed purple complex. Based on this colorimetric phenomenon, CEA concentration in the serum is analyzed by semiquantitative analysis by naked eye and quantitatively analyzed by UV-vis absorption.

## 2. Experimental

### 2.1. Materials and Reagents

Carcinoembryonic antigen (CEA), monoclonal CEA antibody (Ab_1_, 0.1 mg/mL), and polyclonal CEA antibody (Ab_2_, 0.1 mg/mL) were purchased from Sangon Biotech Co., Ltd. (Shanghai, China). Sodium salicylate (C_7_H_5_NaO_3_), N-hydroxysulfosuccinimide sodium salt (NHS), N-(3-dimethylaminopropyl)-N′-ethylcarbodiimide hydrochloride (EDC), melamine, anhydrous ferric chloride (FeCl_3_), ethylene glycol, nitric acid (HNO_3_), hydrochloric acid, sodium acetate, Tween-20, and bovine serum albumin (BSA) were purchased from Aladdin Reagent Company (Shanghai, China). The phosphate buffer solution with various values was prepared with 0.1 M disodium phosphate.

### 2.2. Apparatus

Scanning electron microscopy (SEM) was carried out on a JSM-7100F scanning electron microscope (JEOL, Japan). X-ray powder diffraction (XRD) was tested on a Bruker D8 diffractometer (Germany) using Cu K radiation (40 kV, 40 mA) with a Ni filter. The ultraviolet-visible (UV-vis) absorption spectra were performed with a UV-3900 UV-vis spectrophotometer (Hitachi Co., Japan). Fourier transform infrared spectrum was recorded on FTIR Bruker alpha II (Germany).

### 2.3. Preparation of g-C_3_N_4_ Nanoparticles

Carboxyl-modified g-C_3_N_4_ nanosheets were prepared according to a previous report [28]. Briefly, 5 g of melamine was calcined at 550°C for 4 h in the muffle furnace. After cooling to room temperature, the yellow g-C_3_N_4_ product was ground into powder for further use. Then, 1 g of g-C_3_N_4_ powder was placed into a round-bottom flask with 100 mL of HNO_3_ (5 M), and backwash was performed for 24 h at 125°C. Finally, the product of carboxylate g-C_3_N_4_ was obtained by cooling, centrifugation, and cleaning with deionized water to pH 7.0.

### 2.4. Preparation of g-C_3_N_4_@Fe_3_O_4_ Nanocomposites

g-C_3_N_4_@Fe_3_O_4_ nanocomposites were prepared according to a previous report with minor revision [29]. First, 0.40 g of above g-C_3_N_4_ nanosheets was added into 60 mL of ethylene glycol. Then, 0.65 g of FeCl_3_ was added with ultrasound for 10 min. After 2.60 g of sodium acetate was added, the mixed solution was stirred vigorously for 20 min. Subsequently, the mixture was transferred to a Teflon-lined stainless-steel autoclave and reacted at 200°C for 8 h. After cooling to room temperature, the black product of g-C_3_N_4_@Fe_3_O_4_ was washed with ethanol several times and dried in vacuum at 60°C. To combine polyclone CEA antibody, g-C_3_N_4_@Fe_3_O_4_ nanocomposites were activated by EDC (0.0383 g) and NHS (0.0230 g) and shaked at room temperature for 30 min. Magnetic separation and washing were performed three times, and the conjugation of Ab_2_-g-C_3_N_4_@Fe_3_O_4_ was collected and stored at 4°C for further use. For comparison, Fe_3_O_4_ nanoparticles and Ab_2_-Fe_3_O_4_ conjugation were prepared according to the above steps.

### 2.5. Construction of Colorimetric Immunoassay

**Scheme 1:**
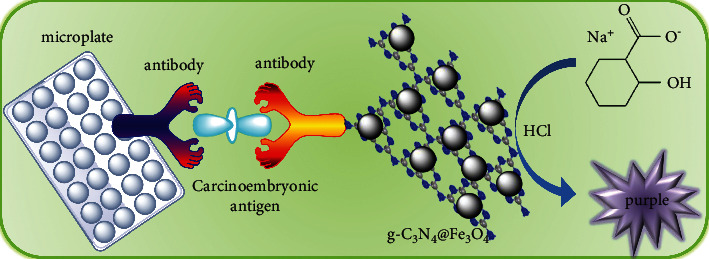
Schematic illustration of colorimetric immunoassay for target CEA detection.

Scheme 1 displays the establishment process of colorimetric immunoassay for CEA detection. First, 100 *μ*L of monoclonal CEA antibody was added into 96-microwell plate, and the plate was covered with plastic wrap and incubated at 4°C overnight. Then, surface solution was removed, and the wells were washed three times with phosphate buffer solution (0.01 M contains 0.05% Tween 20). Then, 100 *μ*L of phosphate buffer solution (0.01 M contains 1% BSA) was added into each well and incubated for 45 min at 37°C to block the nonspecific adsorption sites. After cleaning steps, 100 *μ*L of CEA standards with various concentrations were added into the wells and incubated at room temperature for 45 min. Next, 100 *μ*L of Ab_2_-g-C_3_N_4_@Fe_3_O_4_ was added and incubated at room temperature for 45 min, which designed as Ab_1_/CEA/Ab_2_-g-C_3_N_4_@Fe_3_O_4_. Subsequently, the colorimetric system was constructed by the reaction between sodium salicylate and Fe^3+^ [32]. Briefly, 100 *μ*L of HCl solution (10 M) was added into each well of the above plates. The solution was transferred to a glass test tube containing sodium salicylate (6 mg/mL) to develop color changes. The absorption spectra in the range of 400–700 nm were monitored after reaction. For comparison, Ab_1_/CEA/Ab_2_-Fe_3_O_4_ also was designed according to the above steps.

## 3. Results and Discussion

### 3.1. Characterizations of g-C_3_N_4_@Fe_3_O_4_ Nanocomposites

**Figure 1:**
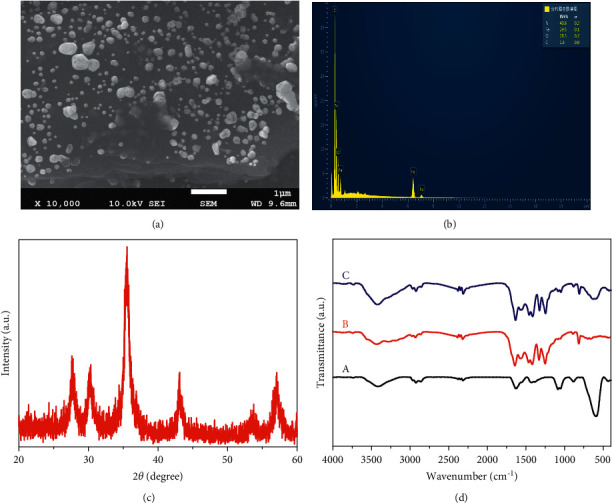
(a) SEM, (b) EDX, and (c) XRD of g-C_3_N_4_@Fe_3_O_4_. (d) FTIR of (A) Fe_3_O_4_, (B) g-C_3_N_4_, and (C) g-C_3_N_4_@Fe_3_O.

The g-C_3_N_4_ nanosheets can load more Fe_3_O_4_ nanoparticles due to its large specific surface area. The morphology of g-C_3_N_4_@Fe_3_O_4_ nanocomposites was characterized by SEM. As shown in Figure 1(a), the Fe_3_O_4_ nanospheres with 200–300 nm is dispersed on the surface of g-C_3_N_4_ nanosheets. Energy dispersive X-ray (EDX) spectra state the elements of Fe, O, N, and C coexisting in g-C_3_N_4_@Fe_3_O_4_ nanocomposites (Figure 1(b)), which preliminarily indicated that the material is successfully synthesized. Furthermore, X-ray diffraction (XRD) is monitored to reveal crystalline structure of g-C_3_N_4_@Fe_3_O_4_ nanocomposite (Figure 1(c)). It can be clearly seen that the peaks at 2*θ* = 30.06°, 35.45°, 40.30°, 53.54°, and 57.16° were assigned to (220), (311), (400), (422), and (511) planes of Fe_3_O_4_, and the peak at 2*θ* = 27.49° was assigned to (002) plane of g-C_3_N_4_. Meanwhile, the function group of g-C_3_N_4_@Fe_3_O_4_ was proved by FTIR spectra.  Figure 1(d) shows the FTIR spectra of Fe_3_O_4_, g-C_3_N_4_, and g-C_3_N_4_@Fe_3_O_4_ nanocomposite materials. Compared with the spectrum of a, b, and c, an apparent band at 3423 cm^−1^ ascribed to O-H stretching vibrations, the band at 595 cm^−1^ attributed to Fe-O vibrations of Fe_3_O_4_, and the band at 800–1600 cm^−1^ attributed to characteristic peak of triazines. These characterizations complement each other and demonstrate the successful synthesis of g-C_3_N_4_@Fe_3_O_4_ nanomaterial.

### 3.2. Mechanism of the Colorimetric Assay

Ferric chloride reagent can react with the phenolic hydroxyl group for color development, which is a classical reaction and is often used to identify drugs, e.g., epinephrine. This reaction is exploited in our color-changing system. g-C_3_N_4_@Fe_3_O_4_ nanolabels can produce Fe^3+^ in acidic conditions, and Fe^3+^ reacts with sodium salicylate for generation of purple complex. This process can be represented by the following equation:

(1) $$\begin{array}{c} {\text{Fe}}_{3}{\text{O}}_{4}+8{\text{H}}^{+}⟶2{\text{Fe}}^{3+}+{\text{Fe}}^{2+}+4{\text{H}}_{2}\text{O,} \end{array}$$

(2) $$\begin{array}{c} {\text{Fe}}^{3+}+{\text{C}}_{7}{\text{H}}_{5}{\text{NaO}}_{3}⟶{\left[\text{Fe}{\left({\text{C}}_{7}{\text{H}}_{5}{\text{O}}_{3}\right)}_{6}\right]}^{3-}+6{\text{Na}}^{+}. \end{array}$$

**Figure 2:**
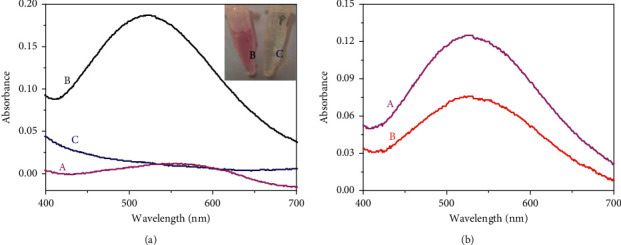
(a) UV-vis absorption spectra of (A) sodium salicylate (6 mg/mL), (B) g-C_3_N_4_@Fe_3_O_4_ + HCl + sodium salicylate, and (C) g-C_3_N_4_@Fe_3_O_4_ + HCl + sodium salicylate + EDTA in 400 nm–700 nm (the inset: photograph of b and c); and (b) UV-vis absorption spectra of (A) Ab_1_/CEA/Ab_2_-g-C_3_N_4_@Fe_3_O_4_ and (B) Ab_1_/CEA/Ab_2_-Fe_3_O_4_ for 1 ng/mL CEA in phosphate buffer solution (pH 7.0).

As shown in Figure 2(a), the sodium salicylate solution has no absorption peak (curve “*a*”), and the purple complex solution owns obvious absorption peaks at 530 nm (curve “*b*”). Whether or not Fe^3+^ has complexed with sodium salicylate, to verify this issue, EDTA is employed to chelate Fe^3+^. Obviously, the purple faded (the inset picture) and the absorption peak disappeared (curve “*c*”). The experimental results verified that the chromogenic mechanism is due to the influence of Fe^3+^.

To highlight the advantages of g-C_3_N_4_@Fe_3_O_4_, two types of colorimetric immunoassays were established based on g-C_3_N_4_@Fe_3_O_4_ and Fe_3_O_4_ nanolabels. At the same conditions, as shown in Figure 2(b), Ab_1_/CEA/Ab_2_-g-C_3_N_4_@Fe_3_O_4_ has a larger absorption value (curve “*a*”) compared with that of Ab_1_/CEA/Ab_2_-Fe_3_O_4_ (curve “*b*”) for 1 ng/mL CEA. This good performance is mainly attributed to the participation of g-C_3_N_4_, which has a large specific surface area and loads more Fe_3_O_4_ nanoparticles.

### 3.3. Performance Assessing of Colorimetric Immunoassay

**Figure 3:**
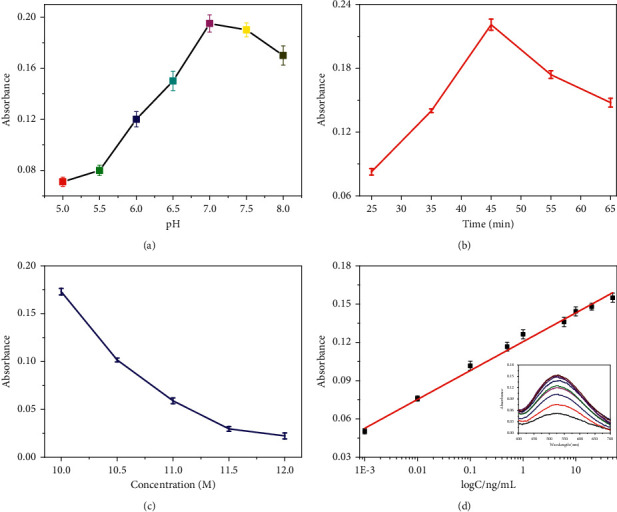
The effect of (a) pH of phosphate buffer solution, (b) incubation time of antibody and antigen, and (c) HCl concentration. (d) Calibration plot of CEA levels (the inset: UV-vis absorption spectrums in 400 nm–700 nm of colorimetric immunoassay toward different CEA concentrations).

For optimal performance of colorimetric immunoassay, experimental conditions related to bioactivity or biosensor sensitivity should be optimized. First, the construction conditions of immune structure including pH and incubation time were optimized. As shown in Figures 3(a) and 3(b), the absorbances have maximum at pH 7.0 and 45 min. Therefore, pH 7.0 and the incubation time of 45 min were used in immunoassay. Meanwhile, the concentration of HCl can resolve Fe_3_O_4_ for producing Fe^3+^, which is directly related to the chromogenic system. As shown in Figure 3(c), there was a maximum at concentration of 10 M, and then, the absorbance gradually declined with the increasing HCl concentration. Therefore, 10 M of HCl was chosen in the whole experiment. Under optimal conditions, CEA was tested by colorimetric immunoassay. As shown in Figure 3(d), the absorbance at 530 nm increased gradually with the increasing CEA concentration in the range of 0.001–50 ng/mL. The linear equation was *y* = 0.022 logC (ng/mL) + 0.120 (*R*^2^ = 0.997, *n* = 27) with the detection limit (LOD) of 0.35 pg/mL (LOD = 3*σ*/*s*, where *σ* is the standard deviation of the blank and *s* is the slope of the calibration plot).

### 3.4. Selectivity, Repeatability, and Stability of the Colorimetric Immunoassay

**Figure 4:**
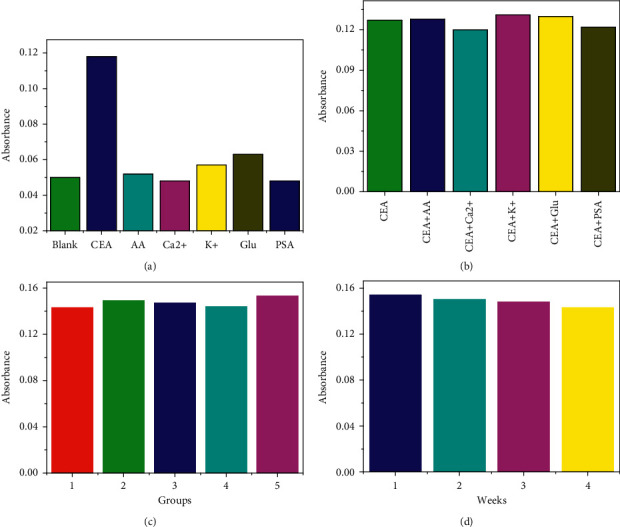
(a) Specificity, (b) antiinterference, (c) reproducibility, and (d) stability of Ab_1_/CEA/Ab_2_-g-C_3_N_4_@Fe_3_O_4_ for 1 ng/mL CEA in phosphate buffer solution (pH 7.0).

In order to ensure the selectivity of colorimetric immunity, some interfering substances were selected for colorimetric detection such as ascorbic acid (AA), Ca^2+^, K^+^, and glucose (Glu) and prostate specific antigen (PSA). As shown in Figure 4(a), the absorbance value of the target CEA is the largest, while that of other interfering substances are smaller. In addition, there was no significant difference in the absorbance values of CEA in the presence of interfering substances (Figure 4(b)). Moreover, five groups of the colorimetric immunosensor were established to test reproducibility. As shown in Figure 4(c), the coefficient of variation (CVs) within the groups was 2.45%. Furthermore, the prepared colorimetric sensor was stored at 4°C for 4 weeks, and its absorbance was measured weekly. Compared with the original absorbance, the value retained 94% (Figure 4(d)). These results indicate that the developed colorimetric immunoassay has high selectivity, good repeatability, and stability.

### 3.5. Actual Serum Sample Analysis

In order to verify the practicability of the colorimetric system in the actual serum matrix, some clinical serum samples were obtained from the First Affiliated Hospital of Shanxi Medical University. Those samples were diluted by phosphate buffer solution (pH = 7.0) and detected by commercial ELISA. The results are given in Table 1, and the *t* test was calculated by the equation: $t_{\exp}=\left({\bar{x}}_{1}-{\bar{x}}_{2}\right)/s\times \sqrt{\left(n_{1}\times n_{2}\right)/\left(n_{1}+n_{2}\right)}$ (where *x* is the average value of three groups of experimental results; *s* is the pooled standard deviation of immunosensor and ELISA toward three groups of experimental results; *n* is the number of analysis (*n* = 3)). It can be seen that all *t*_exp_ values were smaller than *t*_crit_ (*t*_crit_ = 4.30). The result demonstrated that the colorimetric immunosensor is reliable for actual sample detection and own good clinical practical value in future.

## 4. Conclusion

In summary, a novel g-C_3_N_4_@Fe_3_O_4_ nanocomposite-mediated immunoassay was built based on colorimetric effects. Under acidic conditions, g-C_3_N_4_@Fe_3_O_4_ underwent dissociation to produce Fe^3+^, which combines with sodium salicylate to form purple complex. The complex products have a specific absorbance value in the UV-visible absorption spectrum. Thus, the quantitative detection of CEA could be realized by a UV-vis spectrophotometer. This strategy opens a new perspective for the application of colorimetric bioanalysis in the future. Future works should focus on the detection of more biomolecule in serum.

## Figures and Tables

**Table 1:** Comparison of the assay results for human serum specimens by using the developed colorimetric immunoassay and the referenced ELISA method.

Sample	Found by the colorimetric immunoassay (mean ± SD, ng/mL, *n* = 3)	Found by ELISA (mean ± SD, ng/mL, *n* = 3)	*t*_exp_
1	0.94 ± 0.216	1.02 ± 0.463	−0.27
2	10.86 ± 1.809	9.96 ± 0.382	0.84
3	19.92 ± 2.413	20.05 ± 3.082	−0.06
4	31.03 ± 4.031	30.84 ± 3.051	0.07
5	39.22 ± 3.885	39.73 ± 1.007	−0.22
6	46.42 ± 3.395	48.18 ± 2.036	−0.77

## Data Availability

No data were used to support this study.
